# The comprehensive detection of miRNA, lncRNA, and circRNA in regulation of mouse melanocyte and skin development

**DOI:** 10.1186/s40659-020-0272-1

**Published:** 2020-02-03

**Authors:** Zhiwei Zhu, Yueyue Ma, Yuan Li, Pengfei Li, Zhixue Cheng, Huifeng Li, Lihuan Zhang, Zhongwei Tang

**Affiliations:** 1 College of Life Science, Shanxi Agricultural University, Taigu, 030801 China; 2 Department of Veterinary Pharmacology and Toxicology, College of Veterinary Medicine, China Agriculture University, Beijing, 100094 China; 3 College of Animal Science and Technology, Shanxi Agricultural University, Taigu, 030801 China

**Keywords:** miRNA, lncRNA, circRNA, Skin, Melanocyte, Development

## Abstract

**Background:**

Pigmentation development, is a complex process regulated by many transcription factors during development. With the development of the RNA sequencing (RNA-seq), non-coding RNAs, such as miRNAs, lncRNAs, and circRNAs, are found to play an important role in the function detection of related regulation factors. In this study, we provided the expression profiles and development of ncRNAs related to melanocyte and skin development in mice with black coat color skin and mice with white coat color skin during embryonic day 15 (E15) and postnatal day 7 (P7). The expression profiles of different ncRNAs were detected via RNA-seq and also confirmed by the quantitative real-time PCR (qRT-PCR) method. GO and KEGG used to analyze the function the related target genes.

**Results:**

We identified an extensive catalogue of 206 and 183 differently expressed miRNAs, 600 and 800 differently expressed lncRNAs, and 50 and 54 differently expressed circRNAs, respectively. GO terms and pathway analysis showed the target genes of differentially expressed miRNA and lncRNA. The host genes of circRNA were mainly enriched in cellular process, single organism process. The target genes of miRNAs were mainly enriched in chromatin binding and calcium ion binding in the nucleus. The function of genes related to lncRNAs are post translation modification. The competing endogenous RNA (ceRNA) network of lncRNAs and circRNAs displays a complex interaction between ncRNA and mRNA related to skin development, such as *Tcf4*, *Gnas*, and *Gpnms* related to melanocyte development.

**Conclusions:**

The ceRNA network of lncRNA and circRNA displays a complex interaction between ncRNA and mRNA related to skin development and melanocyte development. The embryonic and postnatal development of skin provide a reference for further studies on the development mechanisms of ncRNA during pigmentation.

## Background

Skin development is a complex, multifactorial process. In recent years, more and more attention has been paid to research on skin and embryo development, and its differentiation and regulation-related factors, which has a great implication on clinical treatment. Melanoblasts are derived from neural crest cells during embryonic development. In mouse embryonic day 8.5–9.5 (E8.5–E9.5), melanoblasts are found in truncal region [1–3]. From E10.5, the melanoblasts begin to migrate along a dorsolateral pathway between the ectoderm and the dorsal surface of the somites [3]. At E11.5 melanoblasts start to enter the epidermis. Then, between E15.5 and E16.5, clusters of melanoblasts move into the developing hair follicles, another part melanoblasts remain at the junction of the dermis and the epidermis [4]. The migration phase of melanoblasts depends on a series of paracrine interactions [5].

In recent years, people have been paying more and more attention to the role of non-coding RNA (ncRNA) as a regulators of post-transcriptional gene expression [6]. ncRNAs include microRNAs (miRNAs), long non-coding RNAs (lncRNAs), and circular RNAs (circRNAs) and PIWI-interacting RNA (piRNA), etc [7]. The function of many ncRNAs are unknown, they were previously thought to be transcriptional noise. Recent studies have shown that they control many functions, including epigenetic modifications [8–10], transcriptional and translational regulation, and RNA and protein scaffolds, which represent a new regulatory approach [11, 12].

miRNAs are a highly conserved class of ncRNAs, about 22 nucleotides long, and function by annealing primarily to the 3′untranslated region (3′UTR) of target messenger RNAs to negatively regulate gene expression at the post-transcriptional level [13]. A number of miRNAs studies have been conducted on melanoma. *miR*-*137* and *miR*-*let*-*7b* downregulate *micropthalmia*-*associated transcription factor (MITF)* expression in melanoma cell lines, which are the major regulators of melanocyte development, survival, and function. Also, *miRNA*-*27a*-*3p* inhibits melanogenesis in mouse skin melanocytes by inhibiting the target gene *wnt3* [14]. In the past few years, many miRNAs have been identified in mouse skin tissue. Despite this, their expression characteristics in the skin during the hair follicle cycle are not fully understood and should be further studied, and should thus be further investigated. lncRNAs were first identified in 2002 by analyzing the mouse transcriptome based on functional annotation of 60770 full-length cDNA libraries [15]. lncRNAs were defined as the ncRNAs that are more than 200 nucleotides long and that represent a large and diverse class of ncRNA molecules [16]. *lncRNA SPRIGHTLY* regulates cell proliferation and anchorage-independent colony formation in primary human melanocytes [17]. Knockdown of *lncRNA SPRY4*-*IT1* blocks melanoma cell invasion and proliferation and increases apoptosis [18]. Although a large number of lncRNAs are present in the genome, only a few have been fully verified. Currently, there are few reports on the identification of mammalian skin lncRNA.

circRNAs are a unique class of RNA with a stable structure formed by special loop splicing [19]. Therefore, circRNAs are not degraded by RNase R [20]. Compared to miRNA and lncRNA, circRNA has higher stability and sequence conservation in mammalian cells [19]. There is now an increased appreciation of their important function in gene regulation. However, there are few reports on the function of circRNA related to melanocyte in the skin, and its role in the mechanism of pigmentation remains unknown. Here, we used RNA sequencing (RNA-seq) to analyze differential expression of circRNA between the black coat color skin and white coat color skin of mouse.

In the study, the miRNA, lncRNA, circRNA, expression profile in the black coat color skin and the white coat color skin of mouse will give us a basic understanding of pigmentation in the skin. The GO and KEGG analyses of the target genes of differentially expressed miRNAs and lncRNAs and the host genes of circRNAs will provide a detailed information about the location of these ncRNAs, as well as their function and regulation pathways. This study can elucidate the key ncRNAs in pigmentation and help to find a new regulation mechanism.

## Results

### Statistical analysis of miRNAs data results

**Fig. 1:**
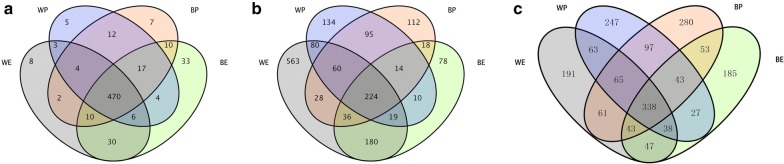
The venn diagram of ncRNAs in two periods. **a** The expression of miRNAs in different periods. **b** The expression of lncRNAs in different periods. **c** The expression of circRNAs in different periods

Venn diagram clearly showed the number of differentiated miRNAs in each stage. In the embryo and postnatal, 470 of the 621 miRNAs all expressed in different coat color (Fig. 1a). For white coat color skin, 483 of 571 miRNAs expressed in white mice at embryonic day (WE) and white mice at postnatal day (WP), 38 miRNAs only expressed in WP, 50 miRNAs only expressed in WE (Fig. 1a). For black coat color skin, 507 of 605 miRNAs all expressed in black mice at embryonic day (BE) and black mice at postnatal day (BP), 25 miRNAs only expressed in BP, 73 miRNAs only expressed in BE (Fig. 1a).

**Fig. 2:**
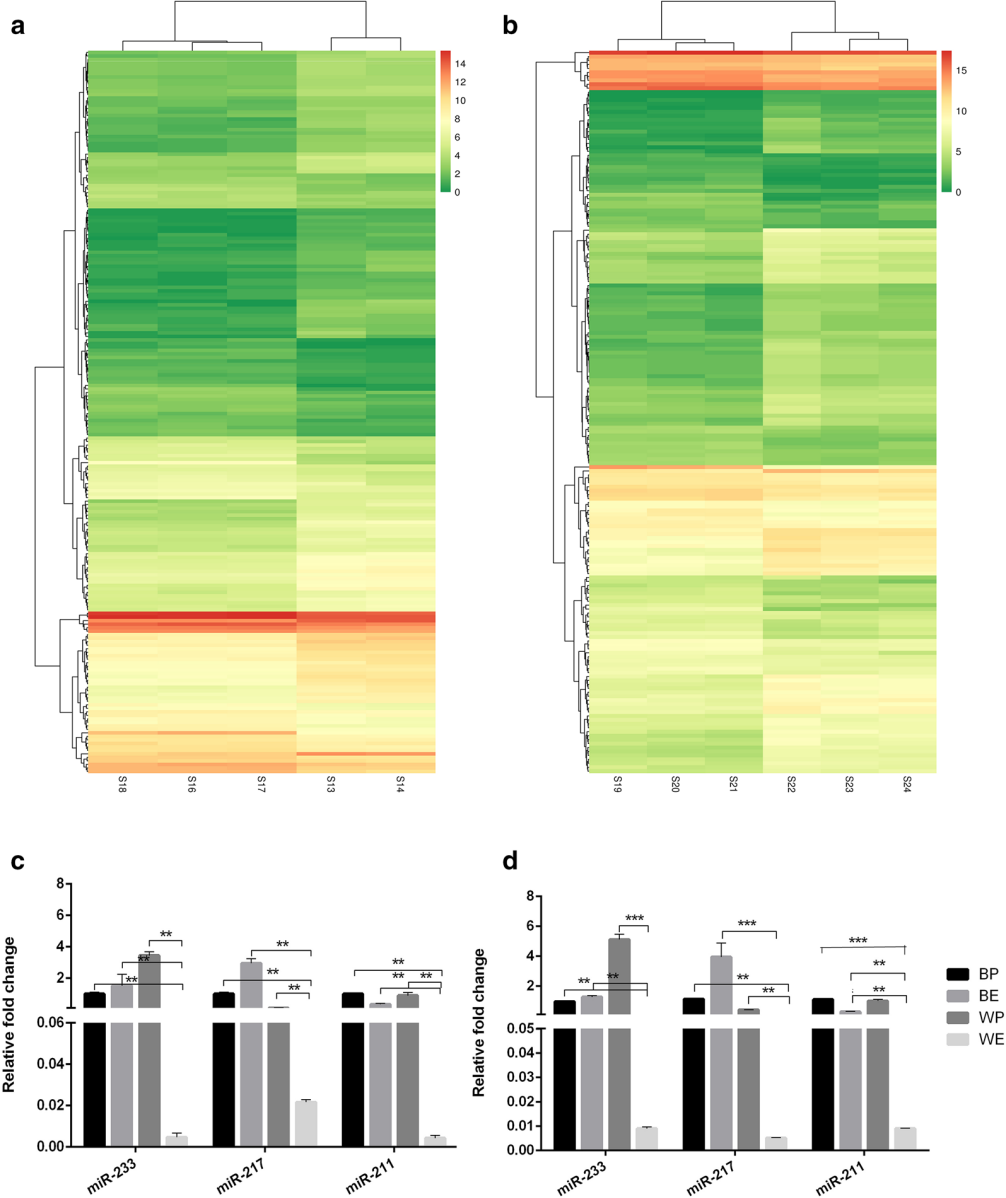
Expression profiles of miRNAs in black coat color skin and white coat color skin during the 15-day embryonic stage and 7-day postnatal stage. **a** Heatmap of differential miRNAs in black coat color skin. The groups 13, 14 were the biological repeat samples of black mice at embryonic day 15, and the groups 16, 17, 18 were the three biological repeat samples of black mice at postnatal day 7. **b** Heatmap of differential miRNAs in white coat color skin. The groups 22, 23, 24 are three biological repeat samples of white mice at embryonic day 15, and the groups 19, 20, 21 are three biological repeat samples of black mice at postnatal day 7. The “S” represents the miRNA sequencing data. **c** Relative expression levels of *miR*-*233*, *miR*-*217* and *miR*-*211* in qRT-PCR. **d** Relative expression levels of *miR*-*233*, *miR*-*217* and *miR*-*211* in RNA-seq

A comparison of the gene expression profile of miRNA in the embryo and postnatal showed that 206 miRNAs were differently expressed in black coat color skin and 183 were differently expressed in white coat color skin (Fig. 2a, b). miRNAs such as *miR*-*233*, *miR*-*217* and *miR*-*211* were chosen based on the significant difference and raw signal intensity of their expressions. *miR*-*233*, *miR*-*217* and *miR*-*211* were not significant difference in BP compared with BE (*P* > 0.05), but were significant difference in WP compared with WE (*P* < 0.001, *P* < 0.01, *P* < 0.01), *miR*-*233* was difference in BP compared with WP (*P* < 0.05), *miR*-*233*, *miR*-*217* and *miR*-*211* were significant difference in BE compared with WE (*P* < 0.01). The result of qRT-PCR of miRNAs were in accordance with the result of the RNA-seq (Fig. 2c, d).

### The function of the target genes of miRNAs

**Fig. 3:**
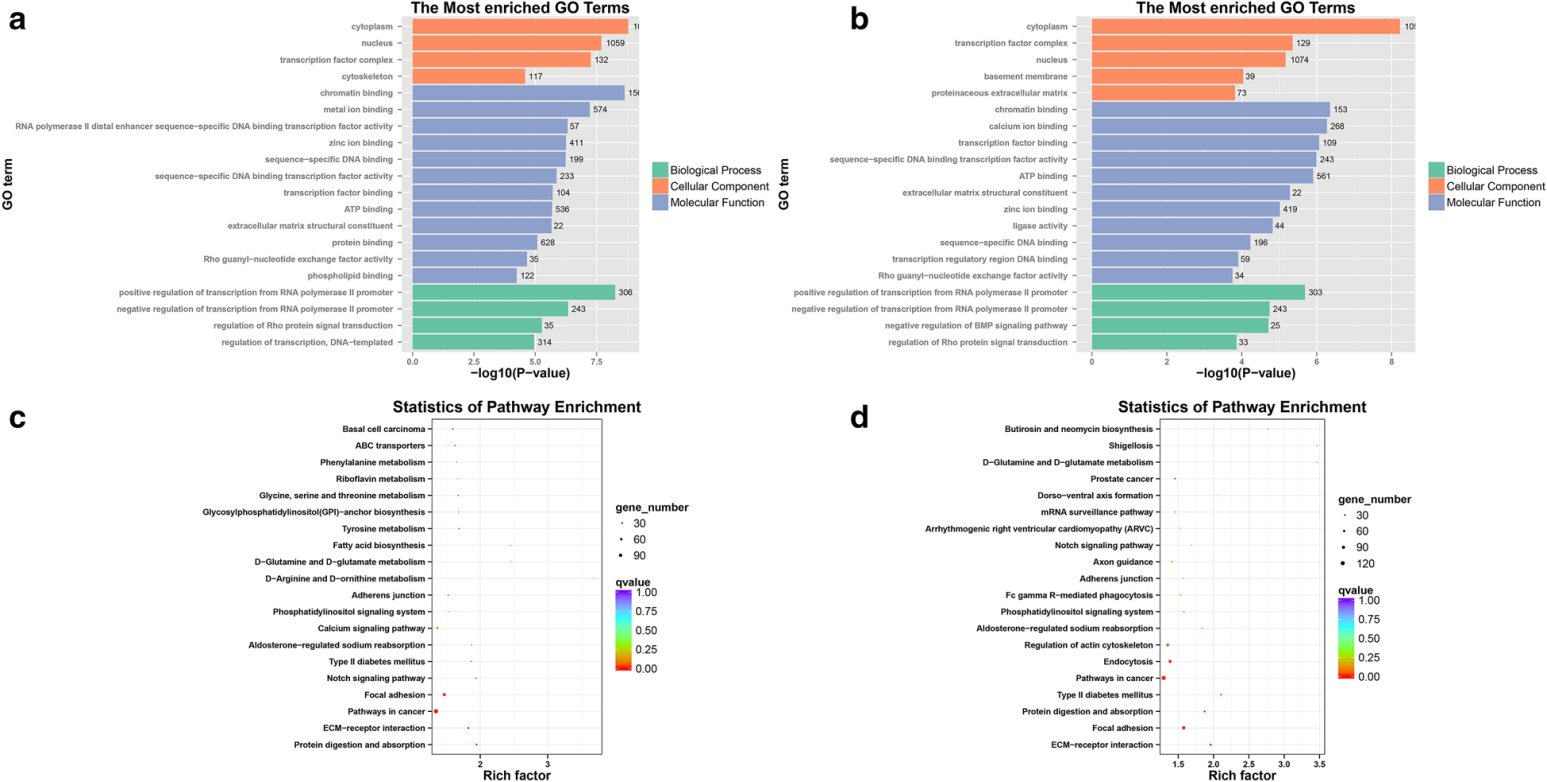
Function analysis of target genes of miRNAs. **a** The most enriched GO terms of target genes of miRNAs in black coat color skin: X axis represents the gene number, Y axis means the GO terms. **b** The most enriched GO terms of target genes of miRNAs in white coat color skin: X axis represents the gene number and Y axis represents the GO terms. **c** Enrichment analysis of target gene KEGG pathway in black coat color skin: X axis represents the enriched factor and Y axis represents the KEGG pathways. The color of the dot indicates the different P-value. The size of the dot indicates the gene number. **d** Enrichment analysis of target gene KEGG pathway in white coat color skin

Gene ontology (GO) analysis was used to analyze the main functions of the differentially expressed miRNAs. According to the GO database, we found that the term enriched by two groups of miRNAs had a high degree of overlap, which indicated that these terms played key roles in embryonic development, including cytoplasm, transcription factor complex and nucleus were in cellular components; chromatin binding, transcription factor binding in black coat color skin. ATP binding and zinc ion binding in the nucleus were in biological processes; regulation of Rho protein signal transduction, positive regulation of transcription from RNA Polymerase II promoter and negative regulation of transcription from RNA polymerase II promoter were in molecular function (Fig. 3a, b).

Through GO enrichment analysis. The enrichment difference between black coat color and white coat color skin mice is mainly reflected in transcription factor binding. Pathway analysis showed the differences are mainly in pathways in cancer, focal adhesion, endocytosis and regulation of actin cytoskeleton (Fig. 3c, d).

### Statistical analysis of lncRNAs data

Venn diagram clearly showed the number of differentiated lncRNAs in each stage. In the embryo and postnatal, 224 of the 1651 lncRNAs all expressed in different coat color (Fig. 1b). For white coat color skin, 383 of 1443 lncRNAs all expressed in WP and WE, 253 lncRNAs only expressed in WP, 807 lncRNAs only expressed in WE (Fig. 1b). For black coat color skin, 292 of 874 lncRNA all expressed in BE and BP, 295 lncRNAs only expressed in BP, 287 lncRNAs expressed in BE (Fig. 1b).

**Fig. 4:**
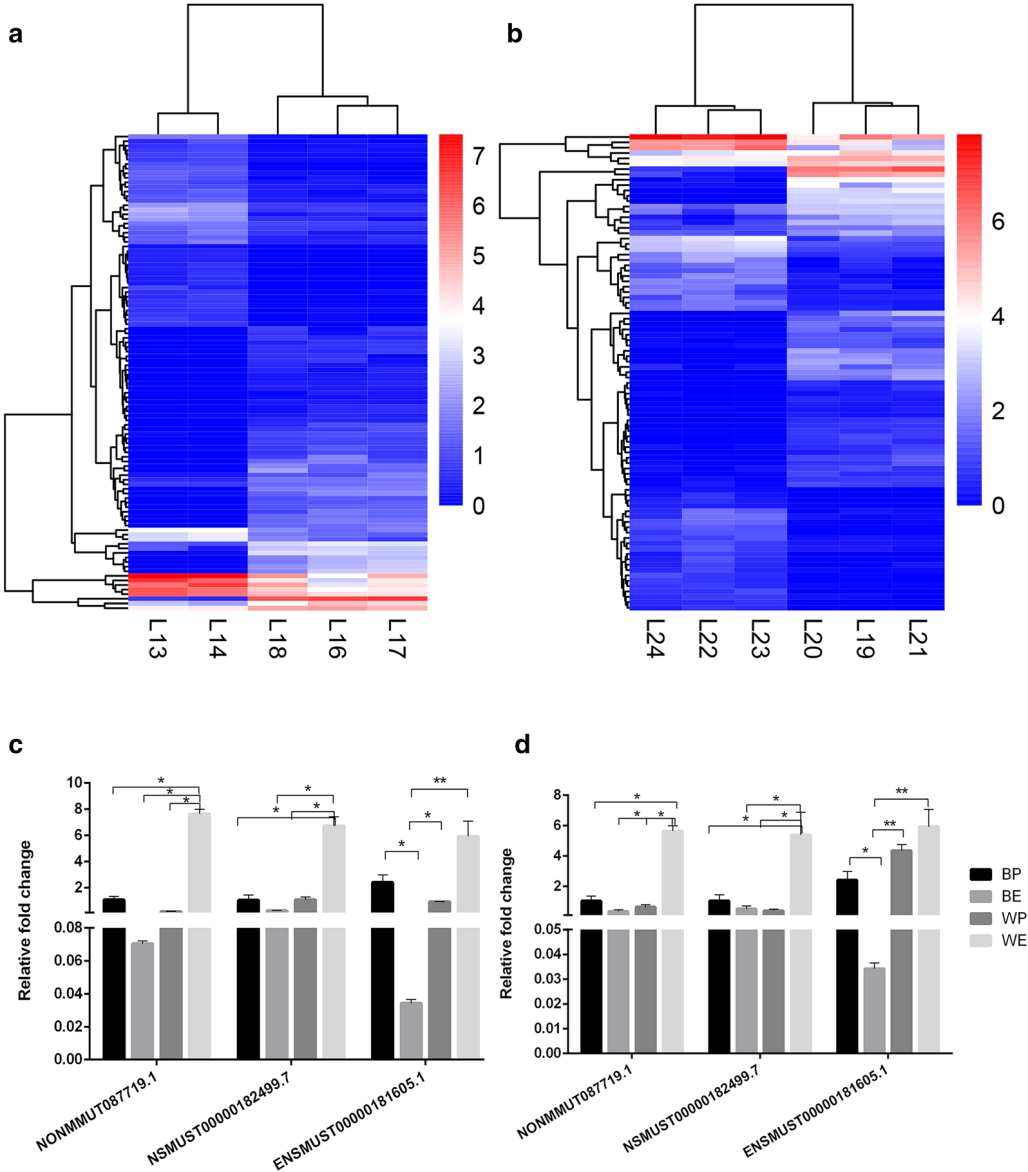
Expression profiles of lncRNAs in black coat color skin and white coat color skin during the 15-day embryonic stage and 7-day postnatal stage. **a** Heatmap of differential lncRNAs in black coat color skin. The groups 13, 14 were the three biological repeat samples of black mice at embryonic day 15, and the groups 16, 17, 18 were the three biological repeat samples of black mice at postnatal day 7. **b** Heatmap of differential lncRNAs in white coat color skin. The groups 22, 23, 24 are three biological repeat samples of white mice at embryonic day 15, and the groups 19, 20, 21 are three biological repeat samples of black mice at postnatal day 7. The “L” represents the lncRNA sequencing data. **c** Relative expression levels of *NONMMUT087719.1*, *NSMUST00000182499.7* and *ENSMUST00000181605.1* in qRT-PCR. **d** Relative expression levels of *NONMMUT087719.1*, *NSMUST00000182499.7* and *ENSMUST00000181605.1* in RNA-seq

A comparison of the gene expression profile of lncRNA in the embryo and postnatal stage showed that 600 lncRNAs were differently expressed in black coat color skin, whereas 813 lncRNAs differently expressed in white coat color skin (Fig. 4a, b). lncRNAs such as *NONMMUT087719.1*, *NSMUST00000182499.7* and *ENSMUST00000181605.1* were chosen based on the significant difference and raw signal intensity of their expressions. *NONMMUT087719.1* and *NSMUST00000182499.7* were not significant difference in BP compared with BE (*P* > 0.05), but *ENSMUST00000181605.1* was difference in BP compared with BE (*P* < 0.05). *NONMMUT087719.1*, *NSMUST00000182499.7* and *ENSMUST00000181605.1* were not significant difference in BP compared with WP (*P* > 0.05), but were difference in BE compared with WE (*P* < 0.05, *P* < 0.05, *P* < 0.01). The result of qRT-PCR of lncRNAs were in accordance with the result of RNA-seq (Fig. 4c, d).

### The function of the target genes of lncRNAs

**Fig. 5:**
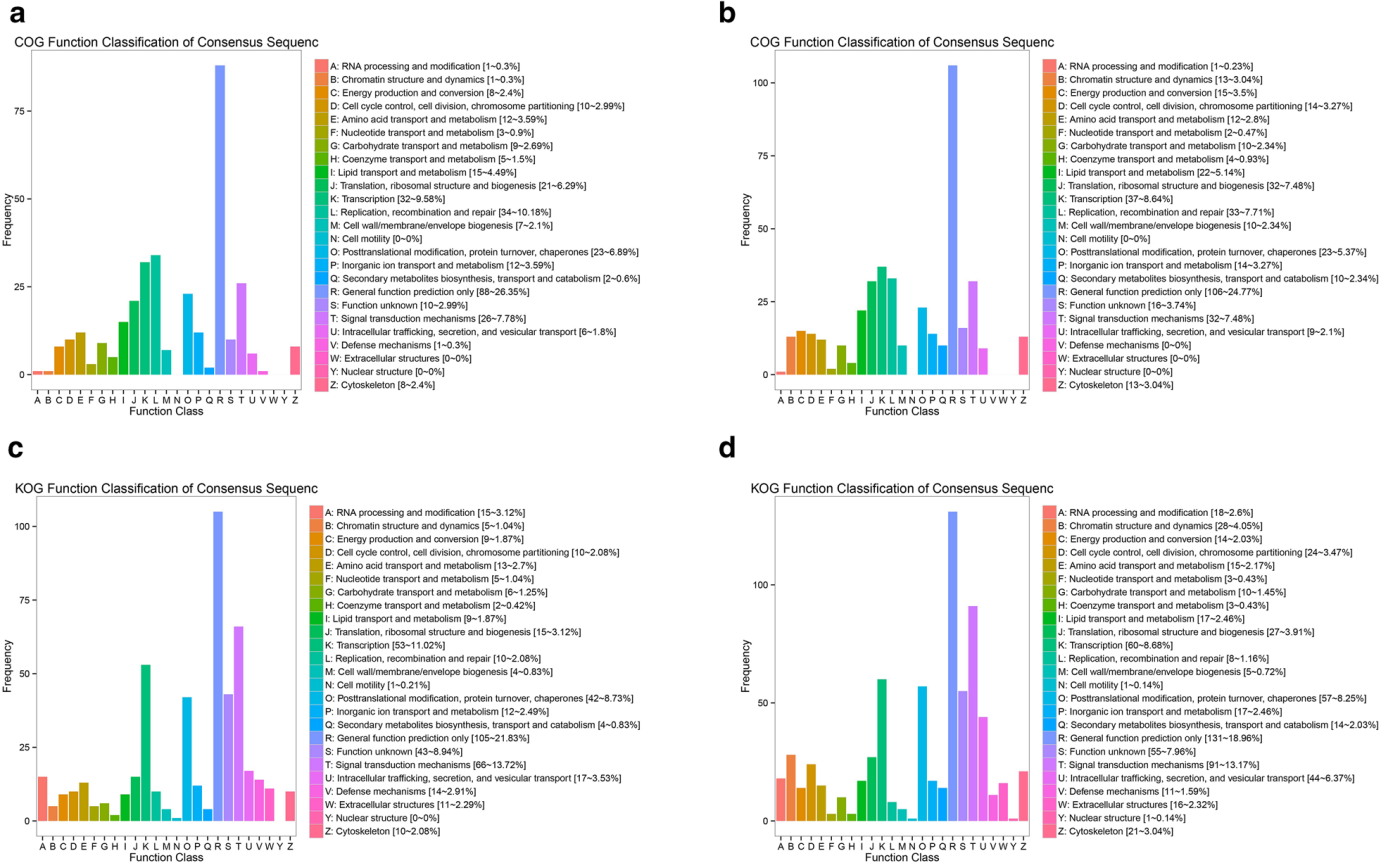
Function analysis of target genes of lncRNAs. **a** The COG analysis of target genes of lncRNA in black coat color skin: X axis represents the COG term and Y axis represents the gene number. **b** The COG analysis of target genes of lncRNA in white coat color skin. **c** The KOG analysis of target genes of lncRNA in white coat color skin: X axis represents the KOG term. Y axis represents the gene number. **d** The KOG analysis of target genes of lncRNA in white coat color skin

Cluster of Orthologous Groups of proteins/Eukaryotic Orthologous Groups of proteins (COG/KOG), and Kyoto Encyclopedia of Genes and Genomes (KEGG) pathway analyses were conducted to explore the function of the related mRNAs. After homologous classification of target genes of lncRNAs, These genes were mainly distributed in 21 protein clusters. The number of genes integrated into protein clusters in white coat color skin was larger than in black coat color skin. These protein clusters mainly included: chromalin structure and dynamics, cell cycle control, cell division, chromosome partitioning, nucleotide transport and metabolism, carbohydrate transport and metabolism, lipid transport and metabolism, translation, ribosornal structure and biogenesis etc. (Fig. 5a–d). KEGG pathways for these genes were mainly enriched in the secondary metabolites biosynthesis, transport, and catabolism in black coat color skin. In white coat color skin, the most enriched KEGG pathways were general function prediction and signal transduction.

### Statistical analysis of circRNAs data

Venn diagram clearly showed the number of differentiated circRNAs in each stage. In the embryo and postnatal, 338 of the 1778 circRNAs were all expressed in different coat color (Fig. 1c). For white coat color skin, 504 of 1260 circRNAs all expressed in WE and WP, 414 circRNAs only expressed in WP, 342 circRNAs only expressed in WE (Fig. 1c). For black coat color skin, 477 of 1277 circRNAs all expressed in BE and BP, 503 circRNAs only expressed in BP, 297 circRNAs only expressed in BE (Fig. 1c).

**Fig. 6:**
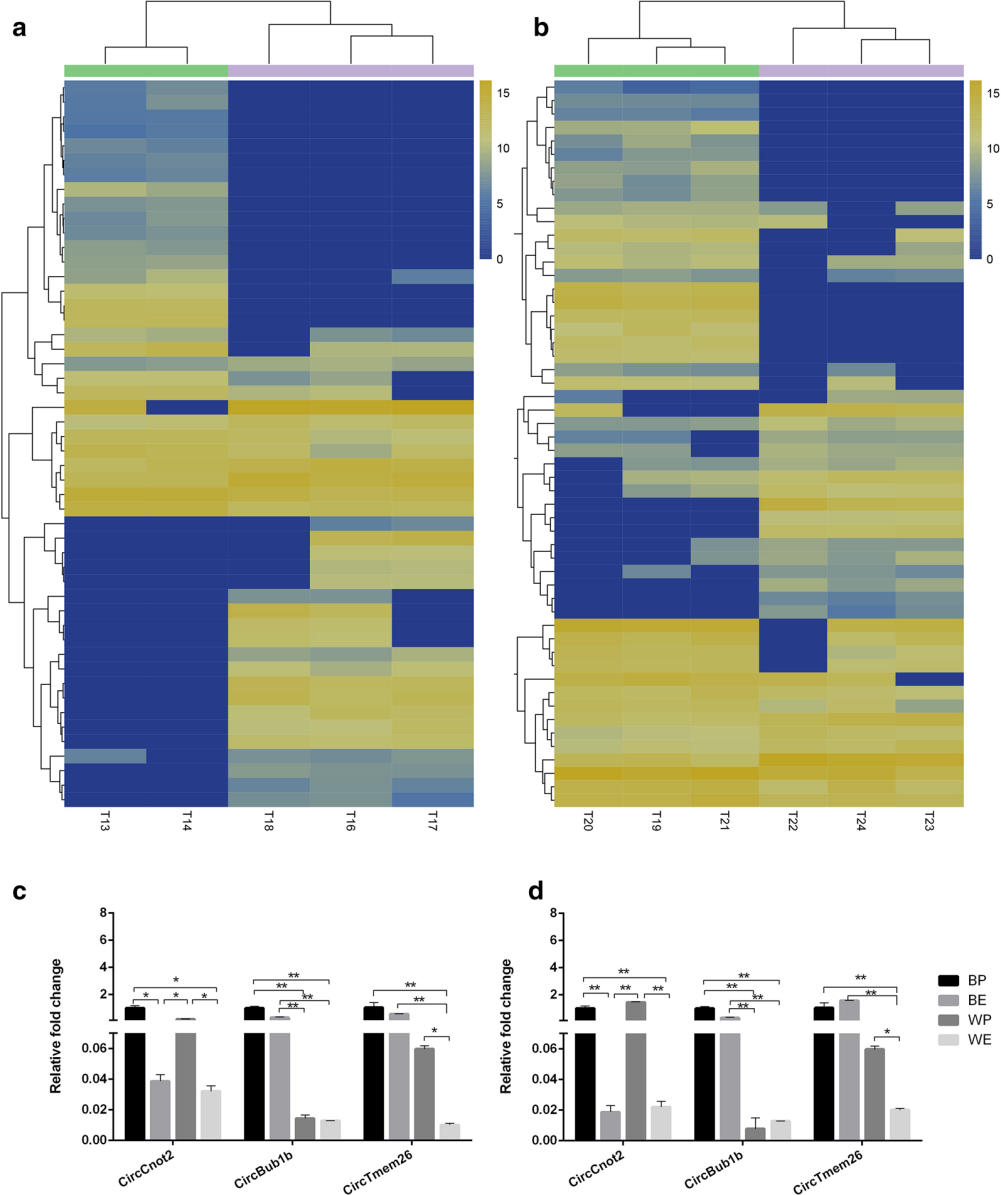
Expression profiles of circRNAs in black coat color skin and white coat color skin during the 15-day embryonic stage and 7-day postnatal stage. **a** Heatmap of differential circRNAs in black coat color skin. The groups 13, 14 were the three biological repeat samples of black mice at embryonic day 15, and the groups 16, 17, 18 were the three biological repeat samples of black mice at postnatal day 7. **b** Heatmap of differential circRNAs in white coat color skin. The groups 22, 23, 24 are three biological repeat samples of white mice at embryonic day 15, and the groups 19, 20, 21 are three biological repeat samples of black mice at postnatal day 7. The “T” represents the circRNA sequencing data. **c** Relative expression levels of *CircCnot2*, *CircBublb* and *CircTmem26* in qRT-PCR.** d** Relative expression levels of *CircCnot2*, *CircBublb* and *CircTmem26* in RNA-seq

A comparison of the gene expression profile of circRNA in the embryo and postnatal showed that 50 circRNAs were differently expressed in black coat color skin, whereas 54 circRNAs were differently expressed in white coat color skin (Fig. 6a, b). circRNAs such as *circConot2*, *circBub1b* and *circTmem26* were chosen based on the significant difference and raw signal intensity of their expressions. *circConot2* was difference in BP compared with BE (*P* < 0.05). *circBub1b* and *circTmem26* were not significant difference in BP compared with BE (*P* > 0.05). *circConot2* and *circTmem26* were difference in WP compared with WE (*P* < 0.05). *circConot2* were not significant difference in BP compared with WP (*P *>* 0.05*), *circBub1b* and *circTmem26* were significant difference in BP compared with WP (*P* < 0.01, *P* < 0.05), *circConot2* was not significant difference in BE compared with WE (*P *>* 0.05*). *circBub1b* and *circTmem26* were significant difference in BE compared with WE (*P* < 0.01). The result of qRT-PCR of circRNA*s were* in accordance with the result of RNA-seq (Fig. 5c, d).

### The function of the host genes of circRNAs

**Fig. 7:**
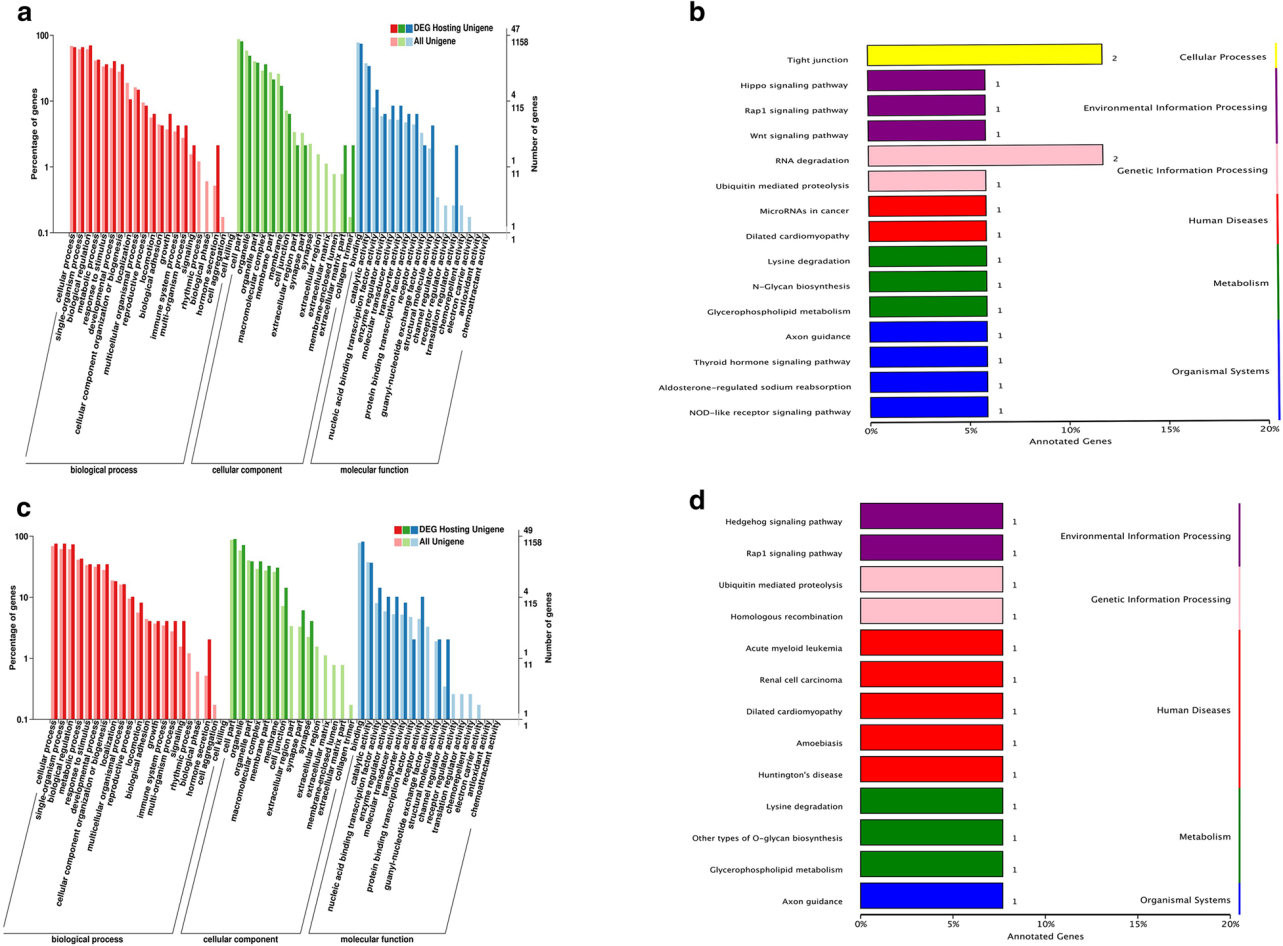
Function analysis of target genes of circRNAs. **a** GO classification of host genes of differential expression circRNAs in black coat color skin: X axis represents the GO terms, Y axis on the left represents the percentage of genes. And Y axis on the right represents the number of the genes. **b** KEGG pathways of the host genes: X axis represents the percentage of the genes and Y axis means the KEGG pathways. **c** GO classification of host genes of differentially expressed circRNAs in white coat color skin. **d** KEGG pathways of the host genes

The function of circRNA is related to the function of the host linear transcripts, then the differentially regulated host genes were further analyzed through GO and KEGG. The differentially expressed genes of black coat color skin enriched in GO terms are extracellular region part, extracellular matrix part, collagen trimer in cellular component. Guanyl-nucleotide exchange factor, activity, structural molecule activity, channel regulator activity, receptor regulator activity and transitioning regulator activity in molecular function (Fig. 7a). Synapse in cellular component, channel regulator activity and receptor activity in molecular function are differentially expressed in the development process of white coat color skin mice (Fig. 7c). Their main differentially expressed genes are at the level of signal conduction and some other cytokines. Through KEGG analysis, the white and black coat color skin mice have common difference signaling pathways including RAP1 signaling pathways, DCM, axon guidance, sugar biosynthesis, lysine, degradation mediated ubiquitin protein hydrolysis, glycerol phospholipid metabolism (Fig. 7d). In the black coat color mice skin have Wnt signaling pathway (Fig. 7b).

### The interplay between the non-coding RNAs

**Fig. 8:**
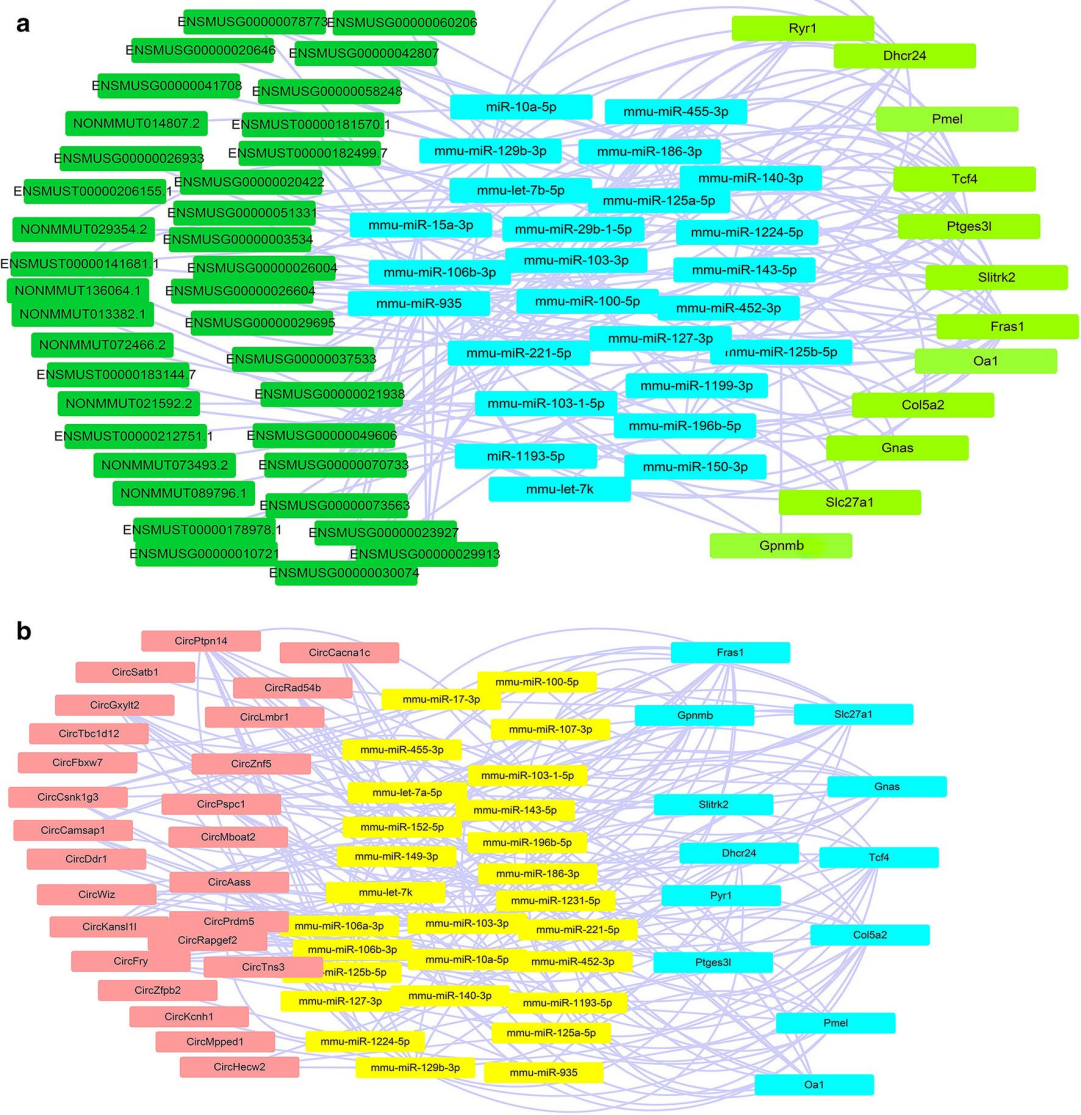
lncRNA-miRNA–mRNA and circRNA–miRNA–mRNA co-expression network. **a** lncRNA–miRNA–mRNA co-expression network. Dark green, blue and pale green are representative of lncRNAs, miRNAs, and mRNAs, respectively. **b** circRNA–miRNA–mRNA co-expression network. Pink, yellow, and blue are representative of circRNAs, miRNAs, and mRNAs, respectively

The network among ncRNA–mRNA was reconstructed via cytoscape. Figure 5a shows that to formulate the lncRNA–mRNA co-expression network used here, we applied Pearson’s correlations to calculate statistically significant associations. The lncRNA–mRNA network was composed of 61 lncRNA nodes, 12 mRNA nodes, and 25 miRNA nodes in the interaction network. The circRNA–mRNA network was composed of 25 circRNA nodes, 12 mRNA nodes, and 27 miRNA nodes (Fig. 3b). mRNAs, such as *transcription factor 4 (Tcf4)*, *pathogen*-*related yeast protein*-*1 (Pry1)*, and *collagen alpha*-*2(V) chain (Col5a2)*, are related to skin development; *ocular albinism type 1 (Oa1), premelanosome protein (Pmel)*, and *Glycoprotein (transmembrane) non*-*metastatic melanoma protein b (Gpnmb)* are related to melanocytes (Fig. 8).

## Discussion

Skin development is a complex multifactorial process. During embryonic development, the differentiation of multipotent progenitor cells in the monolayer epidermis forms the epidermis and its appendages. Melanocytes from neural crest are a pluripotent embryonic cell population and a pigment-producing cell [21]. The neural crest cells migrated from the dorsal to the ventral side around embryonic day 9 (E9) and produced around embryonic day 14.5 (E14.5) [22, 23]. Hair emerges around postnatal day 7 (P7) [24, 25]. These studies demonstrate the feasibility of the transcriptomic analysis of purified melanoblasts at key embryonic day 15 (E15) and key postnatal day 7 (P7), revealing the involvement of previously unreported genes in melanoblast development.

In this study, we provide the expression profile of ncRNAs related to melanocyte and skin development like miRNA, lncRNA, and circRNA in black coat color skin and white coat color skin at E15 and P7. The study identified 206 miRNAs, 600 lncRNAs, and 38 circRNAs in black coat color skin during E15 and P7, and 183 miRNAs, 813 lncRNAs, and 54 circRNAs in white coat color skin during E15 and P7. The results of qRT-PCR are in accordance with the RNA-seq data. This indicates that the data of RNA-seq is valid. GO, COG, KOG, and KEGG analysis showed the function of the differentiated ncRNA related genes and also the location and pathways of the target genes. This gave us a comprehensive understanding of ncRNAs, as well as their location, pathways, and functions.

Recent studies demonstrated that the expression profiles of miRNA showed tissue-specific expression patterns in the epidermis hair follicles, stage-specific expression in the periodic development of hair follicles as well as pigmentation [26–28]. miRNAs in different cell types form a comprehensive, multi-level network system through interactions with signal pathway and regulation factors [29, 30]. In this study, the GO terms of the target genes of differentially expressed miRNAs in black coat color skin are mainly enriched in the nucleus of its cellular component, chromatin binding metal ion binding in molecular function, positive regulation of transcription from RNA polymerase promoter, and negative regulation of transcription from RNA polymerase promoter in biological process. In white coat color skin, the mRNAs related to differentially expressed miRNAs were enriched in the nucleus and in the transcription factor complex in the cellular component, ATP binding and zinc ion binding in molecular function, positive and negative regulation of transcription from RNA polymerase promoter in biological process, which are the same as the black coat color skin group. The enrichment difference between black coat color and white coat color skin mice is mainly reflected in transcription factor binding. It is very important in regulating the transcription of protein in the cell, affecting cell function. The KEGG pathway of pathways in cancer and focal adhesion are both enriched in the black coat color skin and white coat color skin during E15 and P7. These different pathways may be part of the difference in mouse coat color.

At present, there are few reports on skin lncRNA in mammals. For example, 4365 potential intergenic lncRNAs in cow with a piebald phenotype were identified by RNA-seq analysis [31]. Another skin lncRNA catalog was derived from human skin cancer [32], and another skin lncRNAs in goats were reported involvement of in skin pigmentation and development [33]. In this study, the functions of the target genes of differentially expressed lncRNAs in black coat color skin during E15 and P7 were mainly enriched in signal transport and metabolism and post translational modification and signal transduction mechanisms and transcription in white coat color skin. The target genes integrated into protein clusters in white coat color skin was larger than in black coat color skin, and the large part of these proteins play important roles in regulating metabolic processes. These pathways are primarily involved in energy metabolism, and we hypothesize that differences in these proteins often lead to higher fat deposits in white coat color skin mice than in black coat color skin mice. KEGG pathways for these genes were mainly enriched in the secondary metabolites biosynthesis, transport, and catabolism in black coat color skin. In white coat color skin, the most enriched KEGG pathways were general function prediction and signal transduction.

circRNAs like *circRIMS2* and *circCSPP1* play an important role during brain development [34]. However, no studies on the role of circRNAs in skin development have been reported. In this study, the GO terms of the target genes of circRNA host genes in black coat color skin are mainly enriched in cell part and organelle in cellular component, binding and catalytic activity in molecular function, cellular process, and single organism process in biological process. In white coat color skin, the GO terms of host genes are same as the black coat color skin group. Through KEGG analysis, the white and black coat color skin mice have common difference signaling pathways including RAP1 signaling pathways, DCM, axon guidance, sugar biosynthesis, lysine, degradation mediated ubiquitin protein hydrolysis, glycerol phospholipid metabolism. These pathways play important roles in the development. In the black coat color mice skin have Wnt signaling pathway. Abnormal Wnt signaling pathway will lead to abnormal pigmentation and hair regeneration.

The ncRNA expression profile in the skin indicated that ncRNAs play crucial roles in many pathways related to the melanocyte and may pave ways way for devising newer molecular therapeutic options for melanoma by modulating them. This study will conclude with a brief discussion of future directions for ncRNAs studies in melanocyte, such as new approaches to model complex ncRNA expression profile, challenges in ncRNA studies, and the impact of ncRNAs on human diseases, such as melanoma. *miR*-*203* is almost undetectable in mouse E13.5 skin, which is still a single layer of epithelial cells, but is one of the most abundant miRNAs at E15.5, which is the beginning of stratification, indicating that its expression is induced during differentiation and stratification [35]. *miR*-*31* was identified as a key factor in controlling hair cycle-related changes [36]. A recent report showed that *miR*-*125b* is highly expressed in skin stem cells and is significantly reduced in early stem cells progeny [37]. It is indicated that *miR*-*125b* modulates epidermal growth factor receptor (EGFR) activity by targeting *vacuolar protein*-*sorting 4 homolog B (Vps4b)* [38]. In the circRNA interaction network, *miR*-*125b* is predicted to co-express with *Fras1* and also with *circAass* and *circPrdm5*. It was suggested that *Fras1* play critical roles in epithelial-mesenchymal interaction during embryonic development [39]. The network of *circAass*-*miR*-*125b*-*Fras* and *circPrdm5*-*miR*-*125b*-*Fras* need further detection.

Evidence have reported that lncRNAs and circRNAs could bind with miRNAs and act as sponges that could adsorb miRNAs, affecting the expression of related miRNAs and the expression of corresponding target genes. In the analysis of circRNA expression profile in fur, it has been found that 11 downregulated and 32 upregulated circRNAs in the embryo of black fur skin and white fur skin, as well as 21 downregulated and 17 up-regulated circRNA in the postnatal stage. circRNA can play a role in pigmentation of mouse skin hair color through circRNA–miRNA–mRNA network [40]. This article focused on the different expression of circRNAs in different coat color skin during the same mouse development period. In the study, we focused on circRNA–miRNA and lncRNA–miRNA co-expression network of the different mouse development period in different coat color skin. circRNA–miRNA and lncRNA–miRNA co-expression network was built based on the predicted miRNA binding sites and correlations between circRNAs and miRNAs, which was ranked by miRanda database according to the *P*-value of the hypergeometric distribution. In this study, circRNA–miRNA co-expression and lncRNA–miRNA co-expression networks were established to explore the relationships between circRNAs and miRNAs and the relationships between lncRNAs and miRNAs in mouse skin development. But the interaction in lncRNA competing endogenous RNA (ceRNA) network needs further experimentation for confirmation.

## Conclusions

The ceRNA network of lncRNA and circRNA displays a complex interaction between ncRNA and mRNA related to skin development and melanocyte development. The embryonic and postnatal development of skin provide a reference for further studies on the development mechanisms of ncRNA during pigmentation.

## Methods

### Ethic statement

Housing and caring C57BL/6J and ICR mouse in this study were conducted in accordance with the Animal Experimentation Ethics Committee of Shanxi Agricultural University, Taigu, China.

### RNA library construction and sequencing

The triplicate biological replicates skin samples were from C57BL/6J and ICR mice in embryo 15 days and postnatal 7 days. The total RNA from the skin was isolated using TRIzol reagent (Invitrogen, CA, USA) according to the manufacturer’s instructions. The quantity and purity of total RNA were analyzed by using Bioanalyzer 2100 (Agilent, CA, USA). The concentration of RNA samples was analyzed by using Qubit 2.0.

The circRNA library was constructed according to instructions from the NEBNext Ultra Small RNA Sample Library Preparation Kit for Illumina. The linear RNA was digested with Rnase R and the rRNA was removed by rRNA probe. The lncRNA library was constructed via removal rRNA by epicentre Ribo-ZeroTM kit, and lncRNAs and circRNAs were randomly interrupted by adding a fragment reagent. The first strand was synthesized by random hexamers using fragmented lncRNAs and circRNA as templates, and then the second strand of cDNA was synthesized. AMPure XP beads were used to purify the lncRNAs and circRNAs. The end of the DNA was repaired as the blunt end via T4 DNA polymerase and Klenow DNA polymerase, and poly (A) tail was added to the 3′ end for sequencing. The fragment size was then selected by using AMPure XP beads. A second strand containing cDNA was degraded by the USER enzyme. Finally, the lncRNA library and circRNA library were amplified by PCR.

The small RNA library was constructed according to the NEB Next Ultra small RNA Sample Library Prep Kit for Illumina kit, strictly. Approximately 1.5 μg of total RNAs were used to prepare the miRNA library supplemented with water to 6 μL. The library was constructed using the small RNA Sample Pre Kit (Illumina, San Diego, USA). T4 RNA Ligase 1 and T4 RNA Ligase 2 were ligated at the 3′ and 5′ ends of small RNA, respectively.

### Quality control

Raw data (raw reads) of fastq format were firstly processed through in-house perl scripts. In this step, clean data (clean reads) were obtained by removing reads containing adapter, reads containing ploy-N and low quality reads from raw data. And the miRNAs reads were trimmed and cleaned by removing the sequences smaller than 18nt or longer than 30nt. At the same time, Q20, Q30, GC-content and sequence duplication level of the clean data were calculated. All the downstream analyses were based on clean data with high quality.

### Comparative analysis

After obtaining clean reads, sequence alignment was conducted with the reference genome to obtain the location information on the reference genome or gene, as well as the sequence characteristic information unique to sequencing samples. Use BWA (https://github.com/lh3/bwa) [41] for comparison. Three softwares, CIRI (https://jaist.dl.sourceforge.net/project/ciri/CIRI2/CIRI_v2.0.6.zip) [41], find_circ (https://codeload.github.com/marvin-jens/find_circ/zip/v1.2) [42] and CIRCexplorer2 (https://codeload.github.com/YangLab/CIRCexplorer2/zip/master) [43], were selected as tools for predicting circRNA.

The lncRNAs transcriptome was assembled using the Cufflinks (https://codeload.github.com/cole-trapnell-lab/cufflinks/zip/v2.2.1) [44] and Scripture [45] based on the reads mapped to the reference genome. The assembled transcripts were annotated using the Cuffcompare program from the Cufflinks package. The unknown transcripts were used to screen for putative lncRNAs. Three computational approaches include CPC/CNCI/Pfam were combined to sort non-protein coding RNA candidates from putative protein-coding RNAs in the unknown transcripts. Putative protein-coding RNAs were filtered out using minimum length and exon number threshold. Transcripts with lengths more than 200 nt and have more than two exons were selected as lncRNA candidates and further screened using CPC/CNCI/Pfam that have the power to distinguish the protein-coding genes from the non-coding genes. As well as the different types of lncRNAs include lncRNA, intronic lncRNA, anti-sense lncRNA were selected using Cuffcompare.

The miRNA transcriptome use Bowtie tools soft [46], The clean reads respectively with Silva database [47], GtRNAdb database [48], Rfam database [49] and Repbase database sequence alignment [50], filter ribosomal RNA (rRNA), transfer RNA (tRNA), small nuclear RNA (snRNA), small nucleolar RNA (snoRNA) and other ncRNA and repeats. The remaining reads were used to detect known miRNA and new miRNA predicted by comparing with known miRNAs from miRBase (http://www.mirbase.org/) [51]. Randfold tools soft was used for new miRNA secondary structure prediction.

### Quantification of gene expression levels

Cuffdiff (v2.1.1) was used to calculate FPKMs of both lncRNAs and coding genes in each sample [52]. Gene FPKMs were computed by summing the FPKMs of transcripts in each gene group. FPKM means fragments per kilo-base of exon per million fragments mapped, calculated based on the length of the fragments and reads count mapped to this fragment.

miRNA expression levels were estimated for each sample: miRNA were mapped back onto the precursor sequence and read count for each miRNA was obtained from the mapping results.

### Differential expression analysis

Use Venn2.0 to visually plot the resulting data. Differential expression analysis of two conditions/groups was performed using the DESeq R package (1.10.1) [53]. DESeq provide statistical routines for determining differential expression in digital gene expression data using a model based on the negative binomial distribution. The resulting *P* values were adjusted using the Benjamini and Hochberg’s approach for controlling the false discovery rate. Genes with an adjusted *P* value < 0.05 and fold change > 2 found by DESeq were assigned as differentially expressed.

### Quantitative real-time PCR

Expression levels of miRNAs, lncRNAs, and circRNAs were performed by qRT-PCR. Three genes were randomly selected for verification to determine the reliability of the obtained data. Total RNA and total miRNA were reverse transcribed to cDNA using the PrimeScript First Strand cDNA synthesis Kit (Takara, Beijing, China) following the manufacturer’s protocol. qRT-PCR was performed using 2X SYBR green qPCR mix (Takara, Beijing, China) in an ABI 7900HT sequence detection machine (Thermo Fisher Scientific, Inc.). The reactions were incubated at 95 °C for 3 min, followed by 40 cycles at 95 °C for 15 s, and 60 °C for 40 s. Primer sequences for 5 miRNAs, 5 lncRNAs, and 5 circRNAs were designed and synthesized (Table 1). *18S RNA* was used as an internal control of lncRNAs and circRNAs and U6 was used as an internal control of miRNAs. Statistical analyses of the results were performed using the 2^−∆∆CT^ relative quantification.

**Table 1:** The primer of ncRNAs

ncRNAs	Primers (5′–3′)	
*ENSMUST00000181605.1*	F	CTGTATGTCAGATGGTCCGTG
	R	GCCAAGCCAGAGTTACCCA
*NSMUST00000182499.7*	F	AGCAAACAAGCCGTGGAA
	R	TGAGGCAAGTGGAGGAAGG
*NONMMUT087719.1*	F	GCAAGTCACTAGCTTCTTCCTC
	R	GTATCCTGTGAGACGGGTTTA
*CircTmem26*	F	CAAGACCCTGGAGACAGCA
	R	AACGAGGCCAAGGAGACA
*CircBublb*	F	AAAACGCCCTACCTTCCA
	R	TCAACAAGTCCACAGCCTCT
*CircCnot2*	F	AAACCAAGTAATCCACGAGAAG
	R	GACATCTTGAGCCATTTTGAAC
*Mmu*-*miR*-*217*	TACTGCATCAGGAACTGACTGGA	
*Mmu*-*miR*-*211*	TTCCCTTTGTCATCCTTTGCCT	
*Mmu*-*miR*-*223*	CGTGTATTTGACAAGCTGAGTTG	
Reverse primer	CTCAACTGGTGTCGTGGGAGTC	

### Statistical analysis

All samples are performed in triplicate, but the embryonic day 15 is lacked one sample due to the sequencing error. Statistical analysis was performed via Student’s t test to compare the different groups using GraphPad Prism 5.0 (GraphPad Software, La Jolla, CA), while Fisher’s exact test was employed to filter the significant GOs and pathways using R software 3.3.1 (R Development Core Team). Genes with a two-sided *P* value < 0.05 and fold change > 2.0 were regarded as statistically significant genes. The *P* value was false discovery rate (FDR) corrected. In both cases, *P* value < 0.05 (two-tailed) was considered statistically significant. The level of significance was set as *P < 0.05, **P < 0.01, ***P < 0.001. miRNA-mRNA, lncRNA-mRNA, and circRNA-mRNA co-expression networks were constructed by Cytoscape software (version 3.4.0; The Cytoscape Consortium, San Diego, CA, USA).

## Data Availability

The data sets supporting the results of this article are deposited in the National Center for Biotechnology Information (NCBI) repository under SRA (Sequence Read Archives) accession: PRJNA518051 and PRJNA530325. The dataset of the report is available from the corresponding author upon request.

## Abbreviations

RNA-seq: RNA sequencing
E15: Embryonic day 15
P7: Postnatal day
E8.5–E9.5: Embryonic day 8.5–9.5
qRT-PCR: Quantitative real-time PCR
ceRNA: Endogenous RNA
ncRNA: On-coding RNA
miRNAs: MicroRNAs
lncRNAs: Long non-coding RNAs
circRNAs: Circular RNAs
piRNA: PIWI-interacting RNA
3′UTR: 3′untranslated region
*MITF*: *Micropthalmia*-*associated transcription factor*
GO: Gene ontology
COG/KOG: Cluster of Orthologous Groups of proteins/Eukaryotic Orthologous Groups of proteins
KEGG: Kyoto Encyclopedia of Genes and Genomes
*Tcf4*: *Transcription factor 4*
*Pry1*: *Pathogen*-*related yeast protein*-*1*
*Col5a2*: *Collagen alpha*-*2(V) chain*
*Oa1*: *Ocular albinism type 1*
*Pmel*: *Premelanosome protein*
*Gpnmb*: *Glycoprotein (transmembrane) non*-*metastatic melanoma protein b*
EGFR: Epidermal growth factor receptor
*Vps4b*: *Vacuolar protein*-*sorting 4 homolog B*
FDR: False discovery rate
